# A reflective process led by a family physician to develop a renal-protection surveillance tool for HIV patients newly started on dolutegravir

**DOI:** 10.4102/phcfm.v13i1.3088

**Published:** 2021-09-30

**Authors:** Junaid Omar, Izak Loftus, Nabeelah Vallie, Richard B. Whitmore, Gaironesa Solomon, Michelle Powell, Samela Mniki, Mosedi Namane

**Affiliations:** 1 Vanguard Community Health Centre, Western Cape Department of Health, Cape Town, South Africa; 2 Department of Family Medicine, Faculty of Health Sciences, University of Cape Town, Cape Town, South Africa

**Keywords:** HIV, renal protection, health system improvement, South Africa, Dolutegravir

## Abstract

A group of Vanguard Community Health Centre doctors embarked on a Health System’s Improvement (HSI) project with the aim of reducing harm to renal function in patients who were either commenced on or switched to a dolutegravir (DTG)-based antiretroviral therapy (ART) regimen since 2019, when the usual monitoring and evaluation of ART-regimen switches were disrupted by the coronavirus disease 2019 (COVID-19) pandemic. This intended harm-reduction exercise, involving a reflective process that was facilitated by the family physician, led to the development of a *Vanguard Renal Protection Surveillance tool*, which is now used at Vanguard to detect and prevent renal decline.

## Introduction

Vanguard Community Health Centre (VCHC) is a primary care facility in the Western Cape (WC), South Africa (SA). During the first wave of the coronavirus disease 2019 (COVID-19) in 2020, the facility began switching HIV-positive people from an efavirenz-based first line antiretroviral regimen (EFV-BR) to a dolutegravir-based regimen (DTG-BR). Because the COVID-19 demanded additional attention from health care workers (HCWs), the switch lacked the usual rigour in monitoring adverse events that may come with the introduction of new medicines.^1^ Informal and unofficial local feedback from colleagues alleged that there could be serious decline in renal function associated with this DTG-BR. This decline was said not to be related to the known and expected increase in serum creatinine of less than 15% from dolutegravir (DTG),^2^ nephrotoxicity from tenofovir (TDF)^3^ or HIV-associated nephropathy.^4^ The concern of possible nephrotoxicity, associated with this new medication, prompted the authors at VCHC to develop a special surveillance system for detecting early renal impairment, so that if necessary, they could implement earlier remedial actions. The project was led and coordinated by the facility’s family physician (FP), who had experiences of several other WC Health System’s Improvement (HSI) programmes.^5,6,7^

## Development of the surveillance tool for renal function

The aim of the initiative was to develop a surveillance tool that could assist clinicians to prevent and/or detect early renal decline in patients who had been commenced on a DTG-based regimen. The tool was developed through rounds of piloting, reflection and discussions amongst the authors and other practitioners at VCHC (in the main, the nurses). This was also seen as an opportunity to train staff on adverse drug reporting (ADR).

**FIGURE 1:**
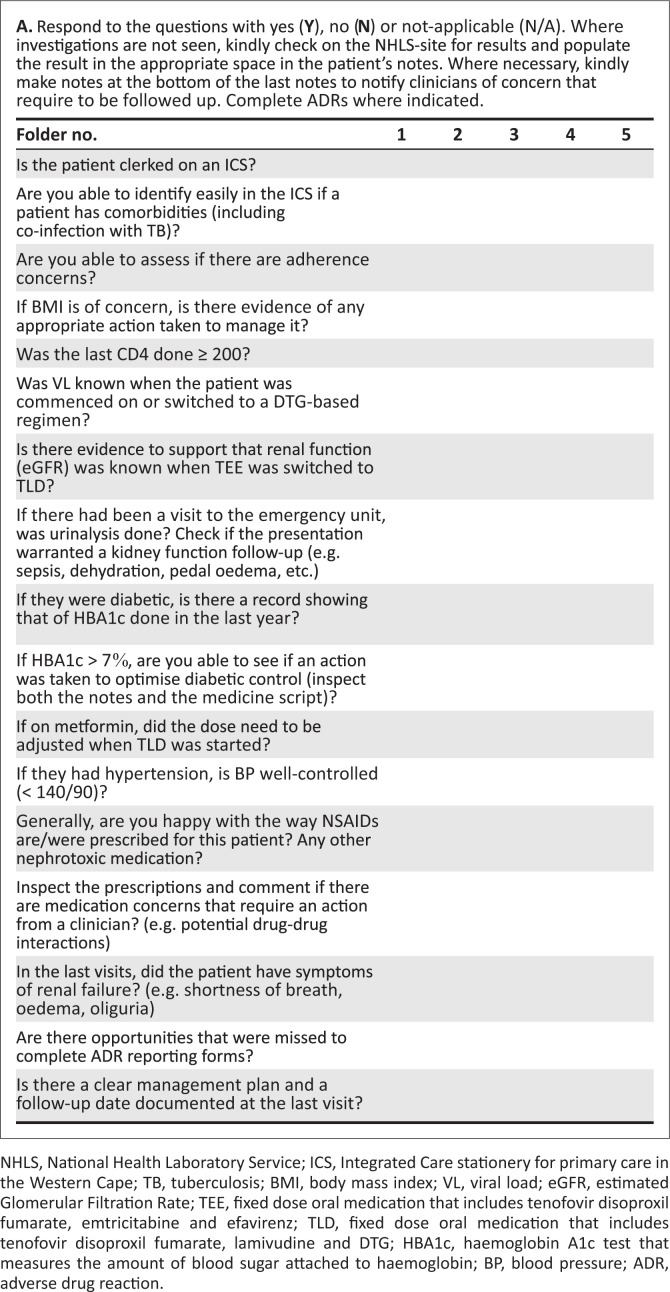
Vanguard renal protection surveillance tool for patients on antiretroviral therapy.

The first task was the drafting of the Vanguard Renal protection Surveillance Tool (VanReST). The tool included potential risk factors for renal disease that had been identified from the SA Primary Health care and Adult Standard Treatment Guidelines.^8,9^ The VanReST (Figure 1) was used to audit folders of HIV-positive people (ranging from children/adolescent ≥ 35 kg and ≥ 10 years to adults), who had been commenced on or switched to a fixed regimen of tenofovir/lamivudine/dolutegravir (TLD) since 2019. The audit intended to review 60 systematic randomly selected folders.

Three medical officer and community-service medical officer pairs were allocated 20 folders to review using VanReST. Protected time was set aside to allow each duo to audit five folders per session, and then reflect on their experience and document their feedback. These reflections were shared with the whole team and were used to refine the tool over several rounds. Once all the folders had been reviewed, the documented feedback was handed in and thematically analysed by the FP and a medical officer. The findings were presented to the HIV unit staff, most of whom had special training in antiretroviral therapy (ART) management and they also gave feedback on the project.

Only 22 folders had a complete set of medical notes. The researchers however, felt that the surveillance tool (Figure 1) was fully developed and reviewing more folders would not refine it further. Table 1 summarises the key themes from the feedback: support for use of tool; poor record keeping as the main barrier to monitor renal function and lack of common understanding of the revised ART protocols with DTG.

**TABLE 1:** Reflections on the project, recommendations, and improvement plans.

Theme	Quote	Recommendations and improvement plans
VanReST was found to be useful in promoting renal health and its continued use was supported	‘From limited data, it seems that there is no increase (in month 4, creatinine compared to baseline level).’ (D2, CS3, M) ‘It helped to create awareness around possible renal impairment in patients on TLD.’ (D3, MO4, F) ‘We need to use this information to implement changes that can address these gaps.’ (D1, MO2, F) ‘Enables us to monitor all the patients on TLD and report any ADRs.’ (D3, MO4, F)	**VanResT** to be adopted at Vanguard and be made a desk-top essential in all consulting rooms. ADR training implemented during the period of study The FP to champion surveillance
A cascade of challenges experienced in locating and/or obtaining complete medical records disrupted continuity of care and made it difficult to do a comprehensive renal-risk surveillance.	‘Procuring the folders has been a challenge.’ (D1, MO1, M) ‘Outside folders were difficult to find.’ (D2, CS2, F) ‘Why are the inners (ART-clinic visit notes) and outers (cover of folder &rest of notes) filed separately?.’ (V, PN1, F) ‘We see patients on what is referred to as duplicates, you cannot call a new folder a duplicate … there is nothing there.’ (V, PN2, F)	Unit manager and clinicians to campaign for a reliable storage and retrieval of folders.
Comments that indicated that there was no common understanding of the latest ART protocols amongst clinicians	‘Also, from my time in ARV clinic, it seems like they aren’t strictly doing renal functions after starting DTG like we expected.’ (D2, MO3, F) ‘After switching from TEE to TLD, the VL, creatinine, eGFR are done routinely, that is, after 12 months and annually as per guidelines. No extra bloods are taken.’ (D3, MO4, F)	SM (our infectious diseases champion) to present updates of the latest ART protocols and ensure every clinician has a correct copy.

D1, reflections from document 1; D2, reflections from document 2; D3, reflections from document 3; V, Verbal comments from stakeholders in a feedback session; CS, community service doctor; MO, medical officer; PN, professional nurse; m, male; F, female; VanReST, Vanguard Renal Protection Surveillance Tool; TEE, fixed dose oral medication that includes tenofovir disoproxil fumarate, emtricitabine and efavirenz; TLD, fixed dose oral medication that includes tenofovir disoproxil fumarate, lamivudine and dolutegravir; DTG, dolutegravir; ADR, adverse drug reaction; ART, antiretroviral therapy; VL, viral load; eGFR, estimated glomerular filtration rate.

## The family physician’s reflections on the journey taken to address an ‘unavoidable’ serious concern

It was necessary to lead by being ‘present’ and not allow the overwhelming COVID-19 pandemic activities to derail the process. The methodology unfolded ‘as we went along’ whilst I held focus for the team. The main challenge encountered at the initial stages was to convince stakeholders that the project was not meant to generate nor analyse quantitative data, but rather to ‘clean up’ the whole system related to renal health.

A bonus of this project has been two-fold: firstly, it gave our young doctors opportunities to experience distributive leadership, to engage in reflective exercises and to advocate for a safe, accessible and continuous healthcare service for HIV-positive people. Secondly, since starting to use the tool, the nurses seem to appreciate the importance of evaluating one’s own practice. They were increasingly reporting on blind spots they identified and gaps that they were closing.

This short report demonstrates how a FP can lead the team in terms of clinical governance to improve quality of care and patient safety. In this example, audit and feedback not only improved care, but also developed a useful tool that could be used going forward. At the same time, the FP capacitated the team of medical officers and nurses to develop skills in quality improvement, monitoring and evaluation. The exercise highlighted the importance of informational continuity that is essential for high quality primary care and the need to implement new clinical guidelines.

## Conclusion

A renal protection surveillance system was developed and implemented at VCHC out of a concern for possibly missing cases of nephrotoxicity during the disruptions of health services during the intersection of the HIV and coronavirus pandemics. The VCHC staff believe that the potential of the surveillance tool will be enhanced by an improved medical record management system. The report illustrates the contribution of FPs to clinical governance and leadership within a primary healthcare team.
